# Supplementary material for: Impaired chloroplast positioning affects photosynthetic capacity and regulation of the central carbohydrate metabolism during cold acclimation
Source: Photosynth Res. 2020 Nov 19;147(1):49–60. doi: 10.1007/s11120-020-00795-y (PMC7728637; doi:10.1007/s11120-020-00795-y)
Supplement: Supplementary file 1 — Supplementary file1 (DOC 5652 kb) [file 11120_2020_795_MOESM1_ESM.docx]

**Supplementary Information**

**
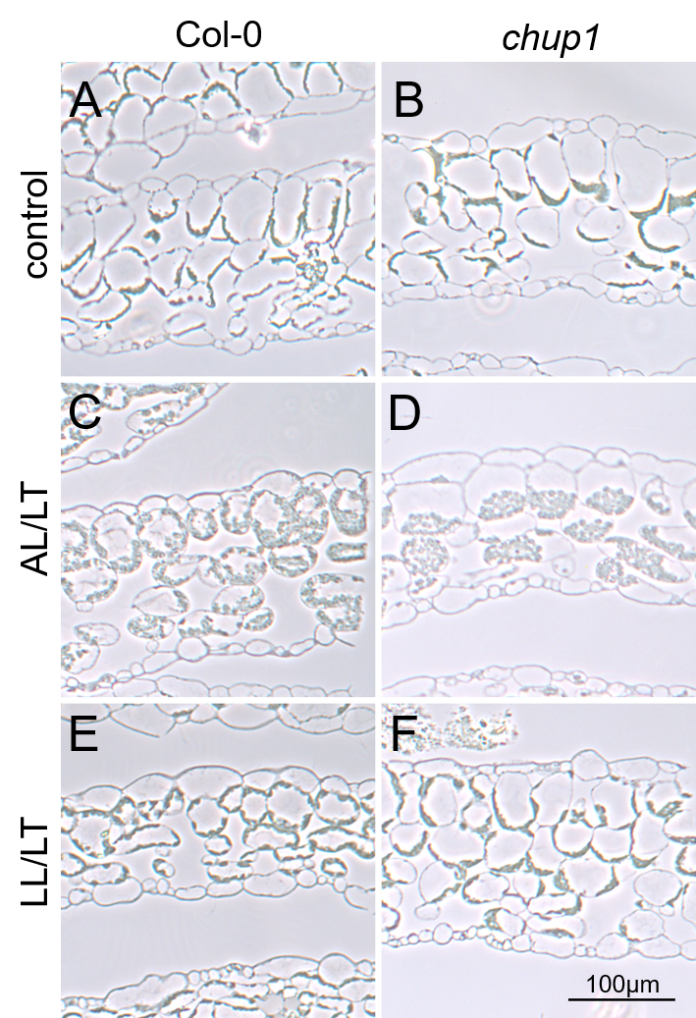
**

**Supplementary Figure S1**. Semi-thin sections of Col-0 and *chup1* primary leaves. (**A, C, E**) Col-0 under control, AL/LT and LL/LT conditions. (**B, D, F**) *chup1* under control, AL/LT and LL/LT conditions. At the end of the night, leaves were kept further 6 hours in darkness to reduce starch content before fixation and ultra-thin-sectioning. Scale bar: 100µm.

**
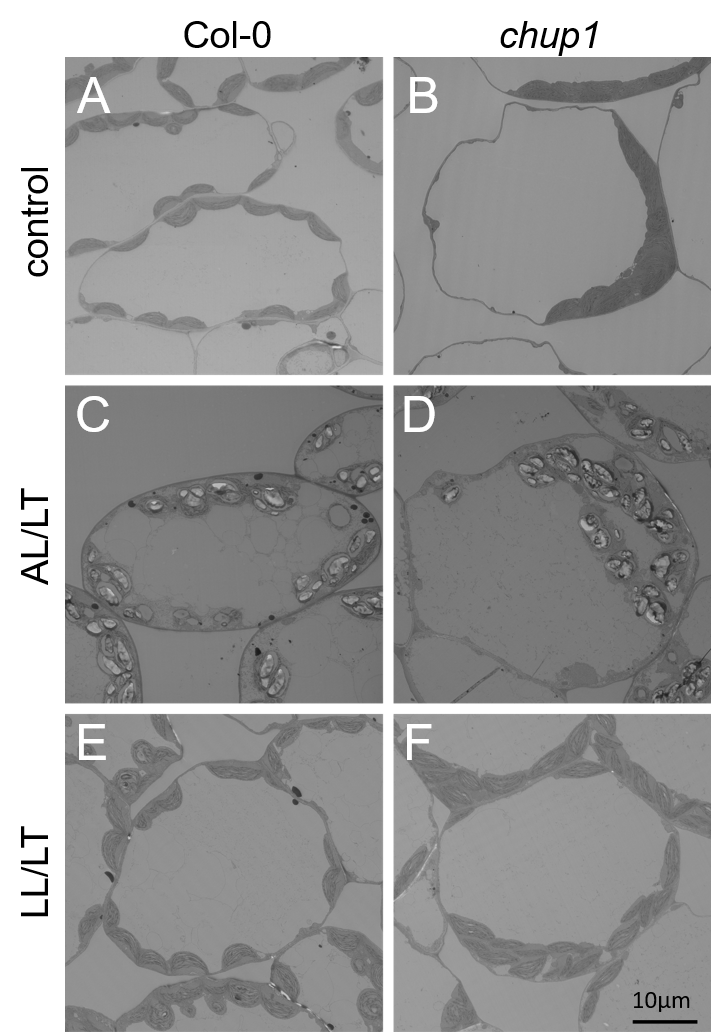
**

**Supplementary Figure S2**. Ultrastructure of mesophyll leaf cells in Col-0 and *chup1* primary leaves. (**A, C, E**) Col-0 under control, AL/LT and LL/LT conditions, respectively. (**B, D, F**) *chup1* under control, AL/LT and LL/LT conditions, respectively. At the end of the night, leaves were kept further 6 hours in darkness to reduce starch content before fixation and ultra-thin-sectioning. Scale bar: 10µm.


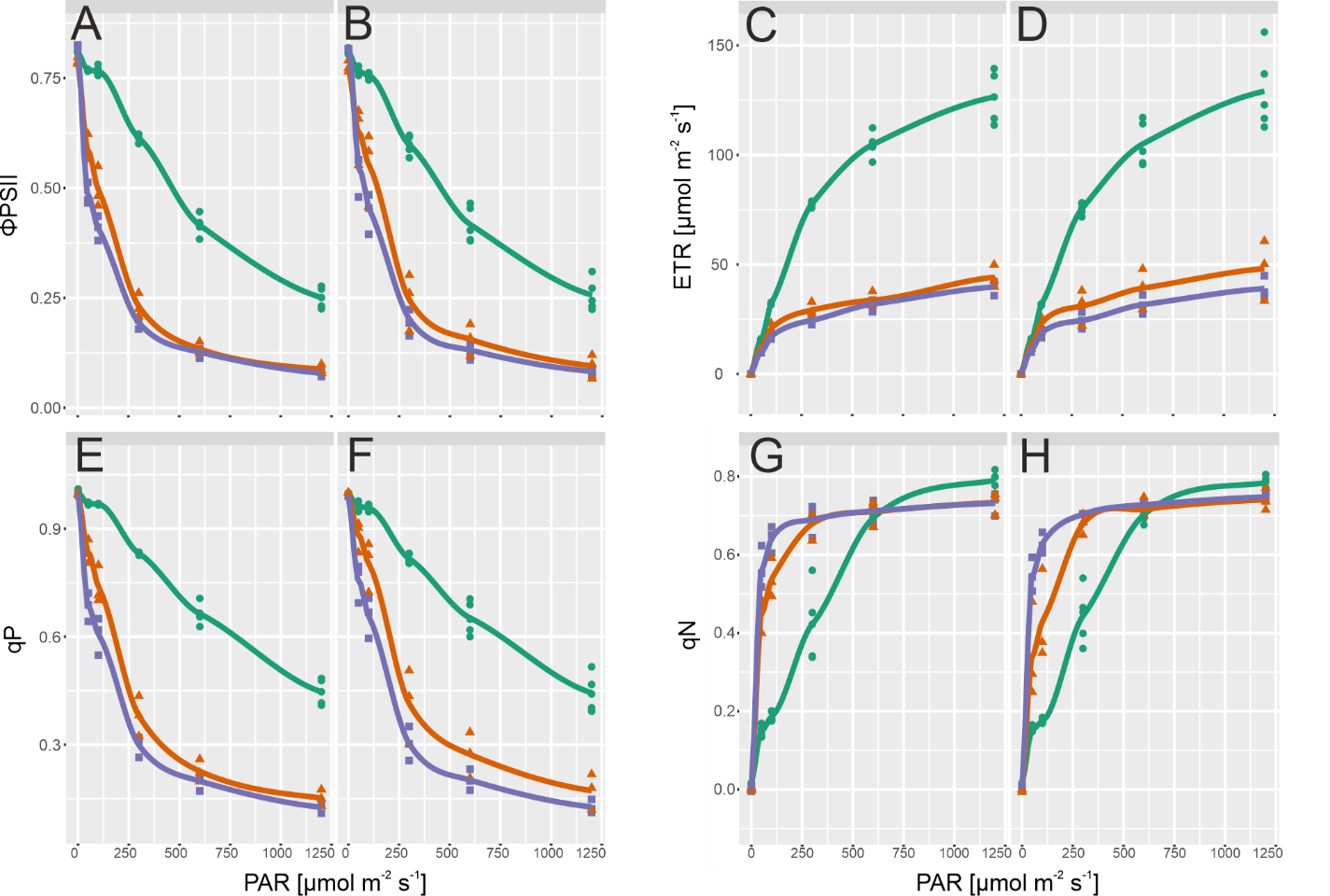


**Supplementary Figure S3. Light response curves of chlorophyll fluorescence parameters.** Effective quantum yield of (**A**) Col-0 and (**B**) chup1. Electron transport rate of (**C**) Col-0 and (**D**) chup1. Photochemical quenching of (**E**) Col-0 and (**F**) chup1. Non-photochemical quenching of (**G**) Col-0 and (**H**) chup1. Green lines and circles: control. Orange lines and triangles: AL/LT. Purple lines and squares: LL/LT. Symbols represent measurements of independent biological replicates (n≥3). Lines represent a local polynomial regression. Measurements of control samples were performed at 22°C, measurements of AL/LT and LL/LT at 4°C. An overview of significances revealed by ANOVA is provided in the supplements (Supplementary Table I).


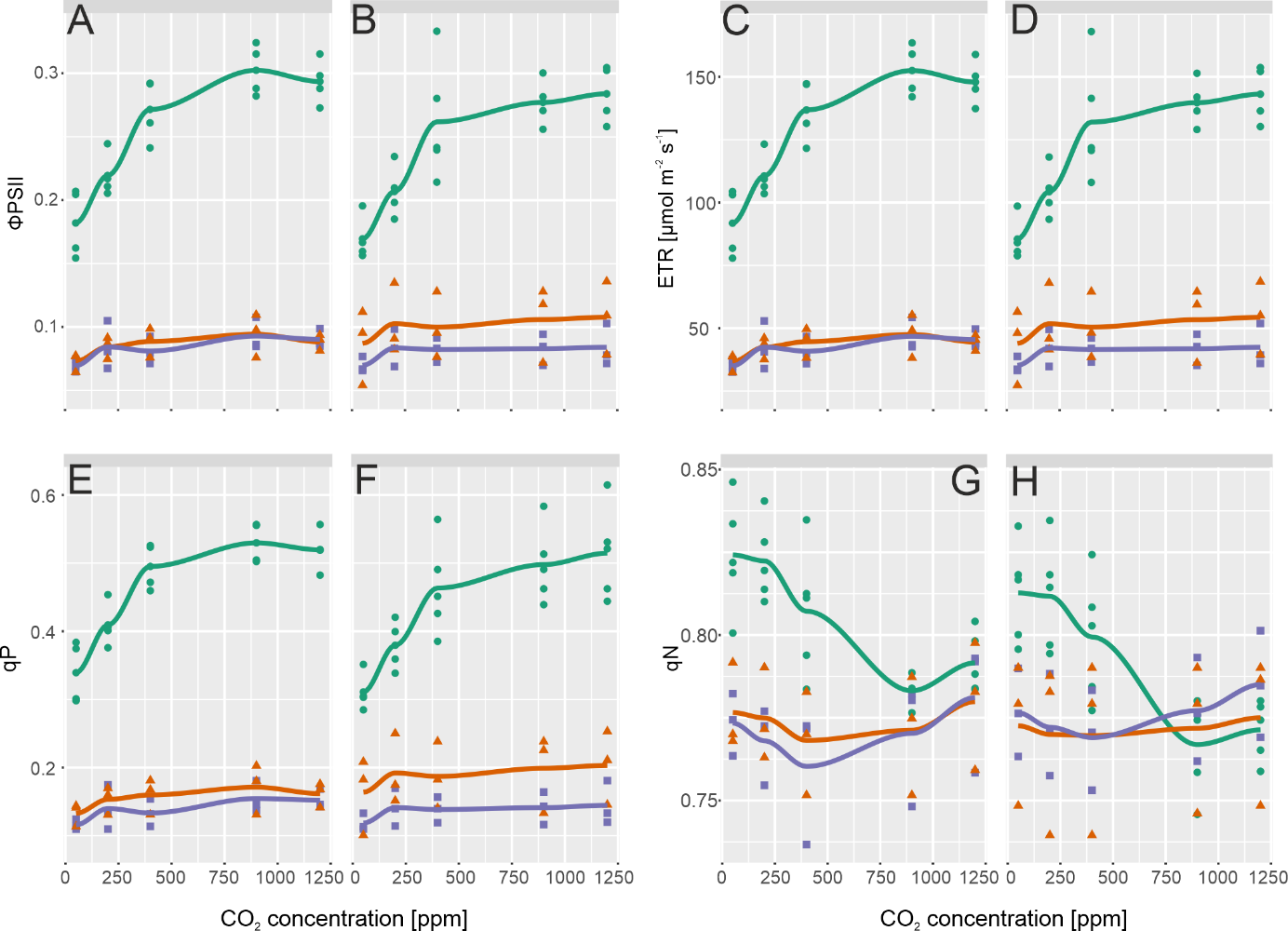


**Supplementary Figure S4**. **CO_2_ response curves of chlorophyll fluorescence parameters.** Effective quantum yield of (**A**) Col-0 and (**B**) chup1. Electron transport rate of (**C**) Col-0 and (**D**) chup1. Photochemical quenching of (**E**) Col-0 and (**F**) chup1. Non-photochemical quenching of (**G**) Col-0 and (**H**) chup1. Green lines and circles: control. Orange lines and triangles: AL/LT. Purple lines and squares: LL/LT. Symbols represent measurements of independent biological replicates (n≥3). Lines represent a local polynomial regression. Measurements of control samples were performed at 22°C, measurements of AL/LT and LL/LT at 4°C. An overview of significances revealed by ANOVA is provided in the supplements (Supplementary Table II).

**Supplementary Figure S6. Schematic structure of the central carbohydrate metabolism.** Arrows represent enzyme reactions which were simulated by solving a kinetic model based on ordinary differential equations (ODEs). Red lines indicate inhibition. Dotted lines indicate lumped reactions. Further information about ODEs, reaction kinetics and parameters is provided in Supplementary Tables IV – VI. F6P: fructose 6-phosphate; G6P: glucose 6-phosphate; 6PGL: 6-phosphogluconolactone; Suc: sucrose; Glc: glucose; Frc: fructose; NPS: net photosynthesis; rStarch: rate of net starch synthesis. rSPS: reaction rate of sucrose phosphate synthase; rINV: reaction rate of invertase; rGLK: reaction rate of glucokinase; rFRK: reaction rate of fructokinase; rPGI: reaction rate of phosphoglucose isomerase; rG6PDH: reaction rate of glucose 6-phosphate dehydrogenase; rEXP: rate of sucrose export to sinks.


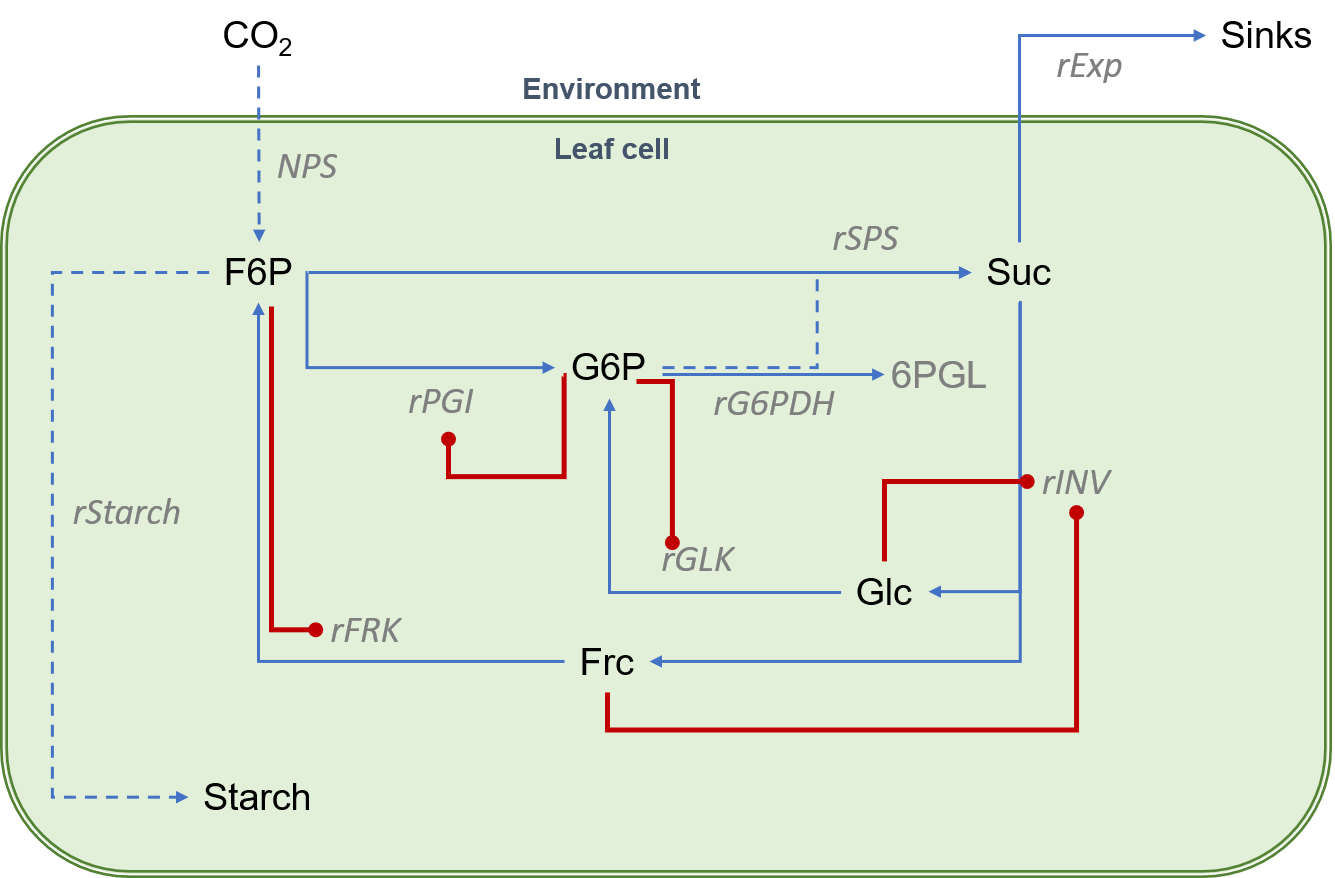


**Supplementary Figure S5. Cold-induced dynamics of PGI and G6PDH activity. (A, C)** enzyme activities in Col-0, **(B, D)** enzyme activities in chup1. Boxes in each panel, left/green: control; middle/orange: AL/LT; right/purple: LL/LT. PGI: phosphoglucoisomerase; G6PDH: glucose 6-phosphate dehydrogenase. Significances are indicated by asterisks but only shown where different between Col-0 and chup1 (ANOVA; ** p <0.01; *** p<0.001). A complete overview of significances is provided in the supplements (Supplementary Table III). n = 5.


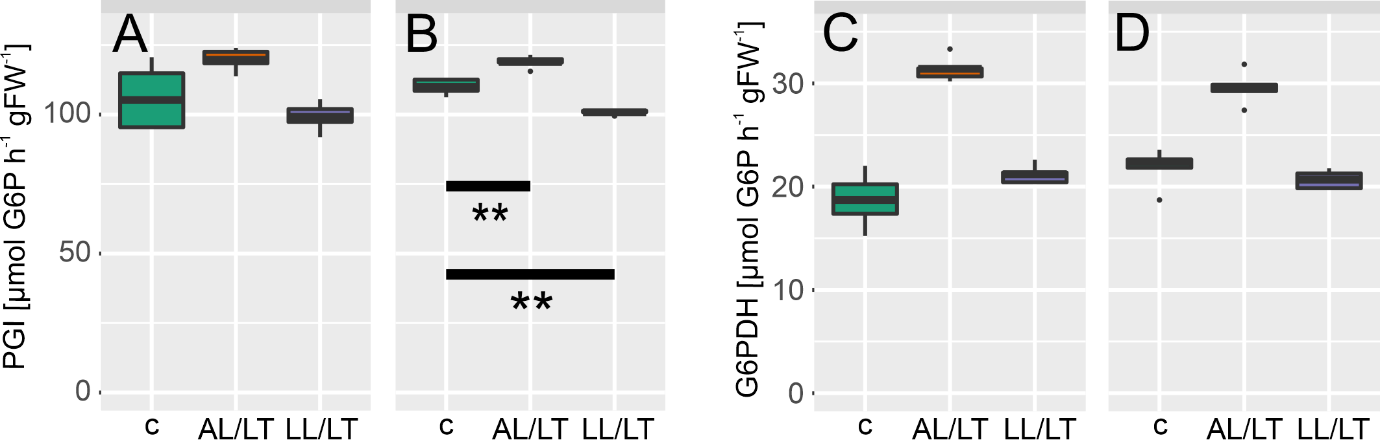


**Supplementary Table I. ANOVA results of photosynthesis light response curves.** c: control; al_lt: ambient light low temperature; ll_lt: low light low temperature. * p<0.05; ** p<0.01; *** p<0.001.

**Supplementary Table II. ANOVA results of photosynthesis CO_2_ response curves.** c: control; al_lt: ambient light low temperature; ll_lt: low light low temperature. * p<0.05; ** p<0.01; *** p<0.001.

**Supplementary Table III. ANOVA results of molecular data.** c: control; al_lt: ambient light low temperature; ll_lt: low light low temperature. * p<0.05; ** p<0.01; *** p<0.001.

**Supplementary Table IV: Ordinary differential equations (ODEs) and enzyme kinetics.** Abbreviations refer to the model structure shown in Supplementary Figure S6.

**Supplementary Table V: Optimized kinetic parameters for steady state simulations in Col-0 and *chup1*.**

**Supplementary Table VI: Range boundaries of model parameters for parameter optimization.** Optimization algorithm: (Global) Particle swarm pattern search (Vaz and Vicente 2007).
